# Risk stratification for early-onset fetal growth restriction in women with abnormal serum biomarkers: a retrospective cohort study

**DOI:** 10.1038/s41598-020-78631-5

**Published:** 2020-12-17

**Authors:** L. Ormesher, L. Warrander, Y. Liu, S. Thomas, L. Simcox, G. C. S. Smith, J. E. Myers, E. D. Johnstone

**Affiliations:** 1 Division of Developmental Biology and Medicine, School of Medical Sciences, Faculty of Biology, Medicine and Health, Manchester Academic Health Science Centre, University of Manchester, Manchester, UK; 2 St Mary’s Hospital, Central Manchester University Hospitals NHS Foundation Trust, Oxford Road, Manchester, UK; 3 Monash University, Scenic Boulevard & Wellington Road, Clayton, 3800 Australia; 4 Department of Obstetrics and Gynaecology, University of Cambridge, Cambridge, UK; 5 NIHR Cambridge Biomedical Research Centre, Cambridge, UK

**Keywords:** Ultrasound, Statistics, Pregnancy outcome, Predictive markers

## Abstract

Abnormal maternal serum biomarkers (AMSB), identified through the aneuploidy screening programme, are frequent incidental findings in pregnancy. They are associated with fetal growth restriction (FGR), but previous studies have not examined whether this association is with early-onset (< 34 weeks) or late-onset (> 34 weeks) FGR; as a result there is no consensus on management. The aims of this study were to determine the prevalence and phenotype of FGR in women with AMSB and test the predictive value of placental sonographic screening to predict early-onset FGR. 1196 pregnant women with AMSB underwent a 21–24 week “placental screen” comprising fetal and placental size, and uterine artery Doppler. Multivariable regression was used to calculate a predictive model for early-onset FGR (birthweight centile < 3rd/< 10th with absent umbilical end-diastolic flow, < 34 weeks). FGR prevalence was high (10.3%), however early-onset FGR was uncommon (2.3%). Placental screening effectively identified early-onset (area under the curve (AUC) 0.93, 95% confidence interval (CI) 0.87–1.00), but not late-onset FGR (AUC 0.70, 95% CI 0.64–0.75). Internal validation demonstrated robust performance for detection/exclusion of early-onset FGR. In this cohort, utilisation of our proposed algorithm with targeted fetal growth and Doppler surveillance, compared with universal comprehensive surveillance would have avoided 1044 scans, potentiating significant cost-saving for maternity services.

## Introduction

Despite the emergence of cell-free DNA testing in 2012, maternal serum biomarker measurement remains a part of aneuploidy screening in many healthcare settings^1–3^. “Extreme” values of these maternal serum biomarkers (defined using multiples of the median) and termed abnormal maternal serum biomarkers (AMSB), are associated with a range of adverse pregnancy outcomes, particularly fetal growth restriction (FGR)^4–8^. AMSB lack sufficient sensitivity to be used in isolation as a primary screening tool for adverse pregnancy outcomes, but evidence-based care pathways for the timing and frequency of surveillance of women with AMSB remain lacking. Currently, there is no consensus on which AMSB should trigger surveillance, what the components of monitoring assessment should be and when and how frequently these assessments should occur. United Kingdom (UK) and New Zealand guidelines recommend serial ultrasound assessment from 26 to 28 weeks’ gestation for low pregnancy-associated plasma protein-A (PAPP-A) only^9,10^, whereas other guidelines are less prescriptive^11^, advising individualised surveillance plans^12^ or varying scan frequency depending on initial ultrasound assessment^13^. Current guidelines also do not delineate the two different phenotypes of FGR: early-onset disease, which occurs between 22 and 34 weeks and is associated with abnormal maternal and fetal placental perfusion, and late-onset disease, characterised by slowing fetal growth after 32 weeks and an absence of measurable placental perfusion defects^14^. Early-onset FGR accounts for ~ 20% of all FGR^15^, but without recognition and intervention, it is associated with a very high stillbirth rate. Although late-onset FGR is also associated with significant risk of poor perinatal outcome^16,17^, adverse outcomes occur much later in pregnancy and the overall stillbirth rate is lower. AMSB are associated with both early- and late-onset FGR^14,15^, however information on the relative distribution of FGR phenotypes within this population is limited. Serial ultrasound assessment of fetal growth can detect both phenotypes of FGR and trigger iatrogenic delivery, but is resource intensive, particularly if frequent serial scans are performed from 26 to 28 weeks. Uterine artery Doppler resistance measurements at 21–24 weeks may improve the ability of ultrasound to detect early-onset FGR^18–22^, but studies using this in AMSB cohorts are small and the numbers of cases of early-onset FGR relatively few^19^. Other investigators have attempted to enhance ultrasound assessment by measuring placental size or volume^19,23–27^, but these techniques have not been widely implemented.

The aim of this study was to determine whether a 21–24 week “placental screen,” comprising ultrasound assessment of fetal biometry, placental biometry and uterine artery Doppler impedance, could identify the subgroup of women with AMSB who were at significant risk of developing early-onset FGR. We also hypothesised that a negative placental screen would be associated with a low probability of early-onset FGR. We aimed to design a model with a high negative predictive value that could be used as a tool to rule-out early-onset FGR without compromising detection rates and therefore direct ultrasound resources more appropriately.

## Results

### Population pregnancy outcomes

Between January 2011 and December 2018, there were 67,065 births at St Mary’s Hospital (SMH), Manchester, UK, of which 65,192 (97.2%) had a complete pregnancy outcome with a birth recorded > 22 weeks’ gestation (Supplementary Table S1). The perinatal death rate for the study period was 6.7/1000 births. SGA affected 12,355 (19.0%) of this population and FGR affected 4491 (6.9%), of whom 427 (0.7%) were born < 34 weeks. Over the same time period there were 29,796 pregnancies in which serum screening was performed, of which 25,688 (86.2%) had a birth > 22 weeks recorded at St Mary’s Hospital. Of the 25,688 pregnancies, 27.4% had combined screening and 12.0% second trimester screening. Amongst the women with abnormal serum markers (1709/25,688 (6.6%)), the prevalence of FGR and early-onset FGR were 12.8% and 2.2%, respectively; these equate to a 2.2- and 5.6-fold increase, compared with the rest of the population. Standard metrics describing the performance of each of the biomarkers at different thresholds in the population data are shown in Supplementary Fig. S1 and Supplementary Table S2.

### Cohort characteristics

**Figure 1:**
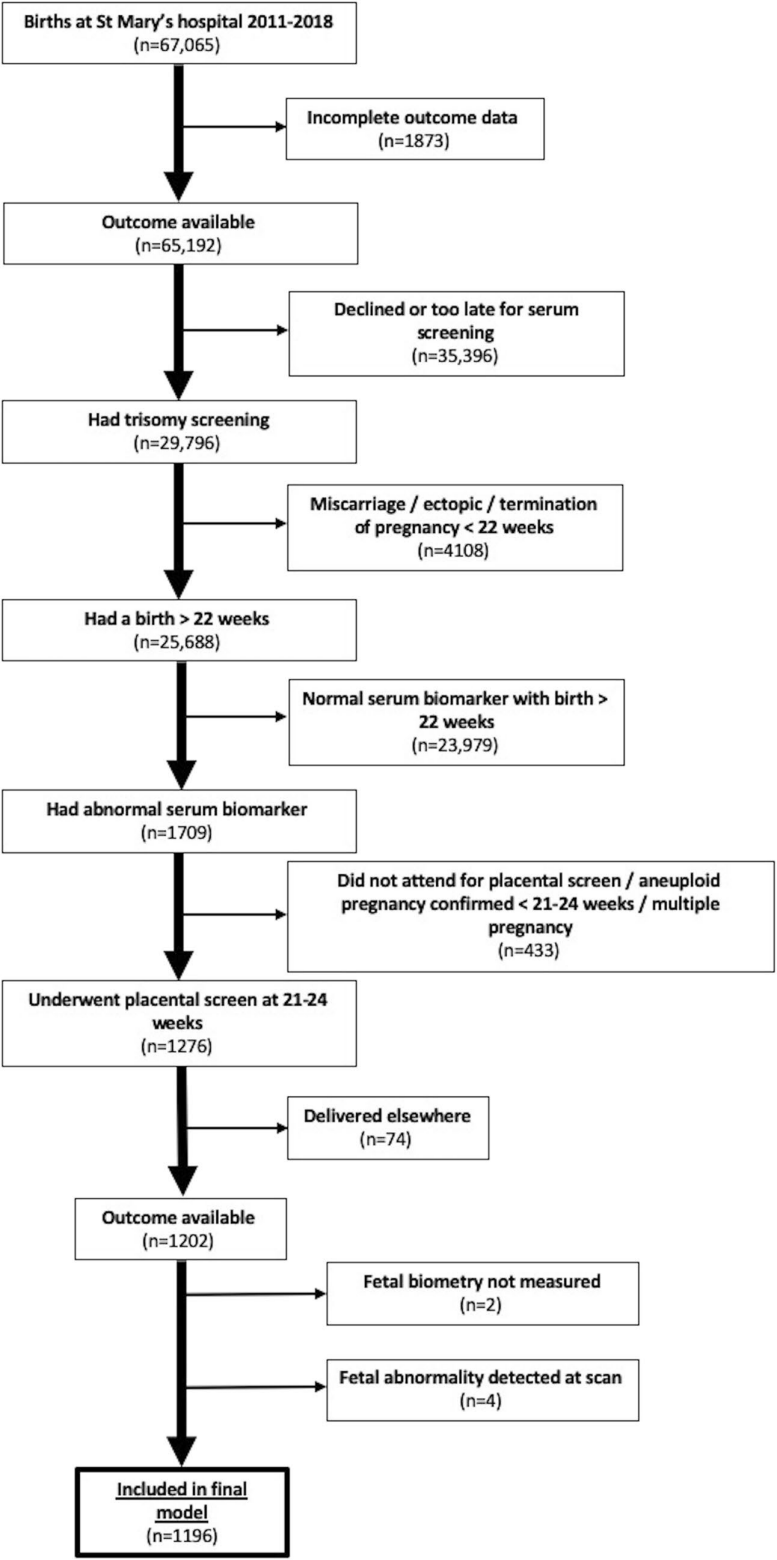
Consort diagram.

1276/1709 (71.0%) pregnancies with AMSB attended at 21–24 weeks’ gestation for a ‘placental screen’. 80 pregnancies were subsequently excluded from the analysis due to incomplete data (n = 74 delivered elsewhere, missing data n = 2) or fetal abnormalities (n = 4) leaving 1196 included (see Fig. 1). Characteristics of the cohort are described in Table 1.

**Table 1:** General characteristics of the study group (n = 1196).

Gestation at assessment* (weeks + days)	23 + 2 (21 + 0–24 + 0)
Gestation at delivery* (weeks + days)	39 + 1 (22 + 6–42 + 3)
**Ethnicity, N (%)**	
White	692 (57.9%)
Black	159 (13.3%)
Asian	211 (17.6%)
Other	134 (11.2%)
BMI (kg/cm^2^)*	25.28 (16.46–54.67)
Delivered < 34 weeks, N (%)	51 (4.3%)
Birthweight* (g)	3145 (300–5119)
Birthweight centile*	29.05 (0·00–100·00)
Birthweight < 10th centile N (%)	293 (24.5%)
Birthweight < 3rd centile N (%)	123 (10.3%)
Early-onset (< 34 weeks) FGR (< 3rd centile/< 10th centile with AEDF) N (%)	27 (2.3%)
Stillbirth, N (%)	12 (1.0%)
Stillbirth < 34 weeks, N (%)	9 (0.8%)

*BMI* body mass index, *FGR* fetal growth restriction, *AEDF* absent end-diastolic flow.

*Median (range) quoted for continuous non-parametric data.

### Cohort pregnancy outcomes

**Figure 2:**
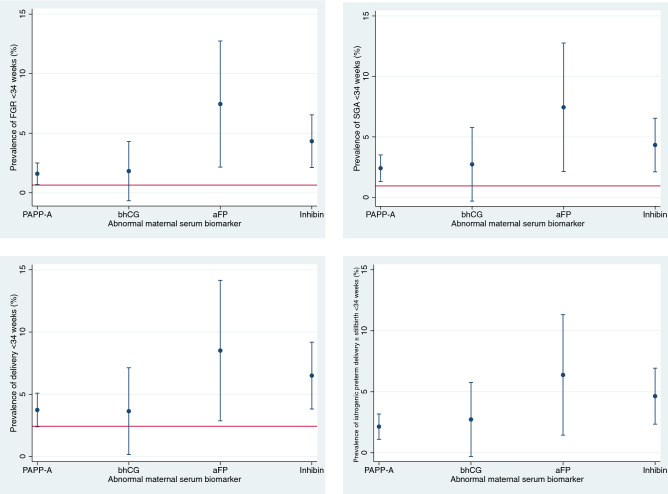
The risk of adverse pregnancy outcomes < 34 weeks associated with different abnormal serum biomarkers. The red horizontal lines indicate the background incidence of each outcome. *Background prevalence of iatrogenic delivery < 34 weeks and stillbirths without congenital anomaly were not reliably coded in electronic health records and therefore has not been included. Illustrated as proportions and 95% confidence intervals.

There was a high rate of SGA (16.4–27.7%) and FGR (7.3–18.1%) across all AMSB (Fig. 2), which was comparable to FGR rates in all women with AMSB in the population dataset (28.9% and 12.8%). The majority (96/123, 78.1%) of cases were late FGR, requiring intervention after 34 weeks. There was a low incidence (27/1196, 2.3%) of early-onset FGR in our study population.

### Statistical modelling

Univariate analysis demonstrated significant associations between early-onset FGR and the following ultrasound parameters: customised estimated fetal weight (EFW) centile, mean umbilical and uterine artery PI and RI, and placental biometry (Supplementary Table S3). Known maternal risk factors for SGA (including ethnicity and parity)^28^ were not predictive of early-onset FGR and were therefore not included in the model.

The best model for exclusion of early-onset FGR (n = 27/1196; 2.3%) included log (customised EFW centile) and log (mean uterine artery PI). This model had a positive likelihood ratio (LR+) of 8.53 and a negative likelihood ratio (LR−) of 0.08 (AUC 0.93, 95% CI 0.87–1.00)). The logistic regression model to calculate the predicted probability of early-onset FGR is as follows:

$$ {\text{Probability score }} = \, \left( {{4}.{3866}0{9} \times {\text{log mean uterine artery PI}}} \right)) - \left( {0.{7}0{89351} \times {\text{log EFW centile}}} \right) - {2}.0{81191}. $$

**Figure 3:**
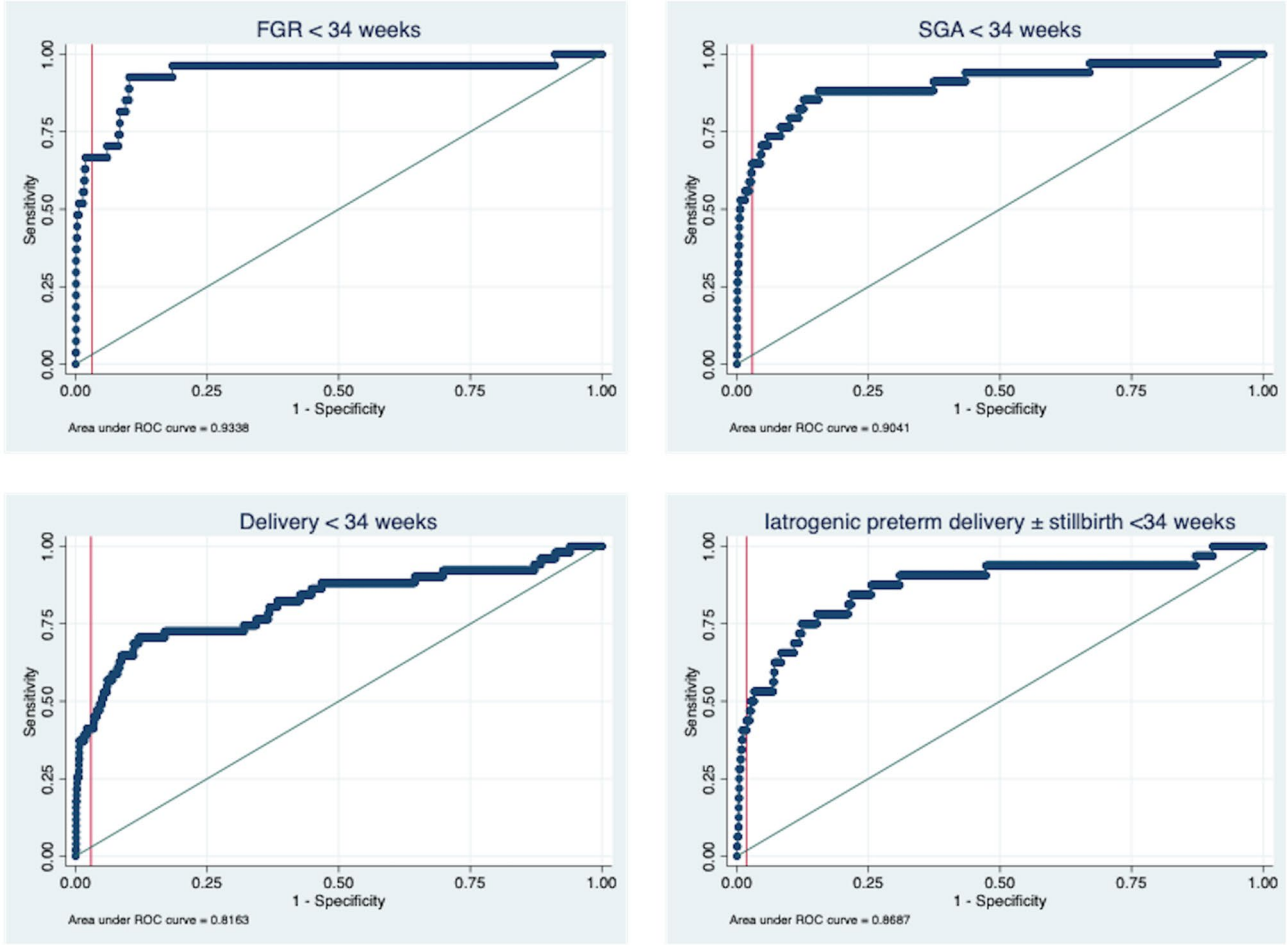
Receiver operating characteristic curve (ROC) analysis of log (mean uterine artery PI) and log (customised EFW centile) to predict adverse pregnancy outcomes < 34 weeks gestation. The vertical lines indicate the threshold for a positive screen.

This combination of log (uterine artery PI) and log (customised EFW) was also predictive of SGA, delivery and indicated delivery < 34 weeks’ gestation (Table 2, Fig. 3). Placental biometry was a significant predictor of early-onset FGR, however inclusion of placental surface area (PSA; width × width) in the model did not significantly improve its performance, despite a halving of the negative likelihood ratio (Supplementary Table S4; p = 0.06 (DeLong); LR+ 9.38, LR− 0.04; AUC 0.94 (95% CI 0.88–1.00)). Additionally, use of population centiles or Z scores for EFW did not improve the model (p = 0.63, AUC: 0.92 (95% CI 0.84–1.00) and p = 0.73, AUC 0.93 (95% CI 0.86–1.00), respectively (DeLong)). Since placenta-mediated FGR is typically asymmetrical, we tested inclusion of a measure of asymmetry (Z score of head circumference/abdominal circumference divided by the Z score of femur length)^29^. This had inferior performance, compared with customised estimate fetal weight centile (p = 0.02, AUC = 0.87 (95% CI 0.79–0.95). The model performance was not different if early-onset FGR was defined using non-customised centiles (28/1198; either with (AUC 0.89 (95% CI 0.81–0.98)) or without (AUC 0.89 (0.79–0.87)) customisation of EFW).

**Table 2:** 21–24 week placental screen test performance for adverse pregnancy outcomes before 34 weeks gestation.

Adverse pregnancy outcome < 34 weeks	True + ve/false −ve	False + ve/true −ve	Sensitivity (95% CI)	Specificity (95% CI)	LR+ (95% CI)	LR − (95% CI)	DOR (95% CI)
FGR (< 3rd centile/< 10th centile with AEDF)	25/2	127/1042	92.6 (76.6–97.9)	89.1 (87.2–90.8)	8.53 (7.01–10.37	0.08 (0.02–0.32)	102.56 (24.01–438.10)
SGA (< 10th centile)	30/4	184/978	88.2 (73.4–95.3)	84.2 (82.0–86.1)	5.57 (4.65–6.68)	0.14 (0.06–0.35)	39.86 (13.88–114.50)
Delivery < 34 weeks	39/12	394/751	76.5 (63.2–86.0)	65.7 (62.8–68.3)	2.22 (1.87–2.64)	0.36 (0.22–0.59)	6.20 (3.21–11.97)
Iatrogenic delivery/stillbirth < 34 weeks	29/3	363/801	90.6 (75.8–96.8)	68.8 (66.1–71.4)	2.91 (2.53–3.34)	0.14 (0.05–0.40)	21.33 (6.46–70.48)

+ *ve* positive, *− ve* negative, *CI* confidence interval, *LR +*  positive likelihood ratio, *LR−* negative likelihood ratio, *DOR* diagnostic odds ratio, *FGR* fetal growth restriction, *AEDF* absent end-diastolic flow, *SGA* small for gestational age.

The regression model characteristics are summarised in Supplementary Table S5. This model was significantly better at predicting early rather than late FGR (AUC 0.70 (95% CI 0.64–0.75)). Using a threshold of ≥ 0.031 to define a “positive placental screen” to compare groups, there was a significant difference in birthweight centiles between the “negative” (1044/1196; 87.3%) and “positive” (152/1196; 12.7%) placental screen groups: median 31.56 (interquartile range 45.27) vs. 6.20 (interquartile range 30.32) respectively, p < 0.001 (Supplementary Table S6, Supplementary Fig. S2). A higher proportion of the “positive” placental screen group delivered before 34 weeks (22.4% compared with 1.6%, p < 0.001) and before 36 weeks (32.9% compared with 5.5%, p < 0.001), with a significant difference in the median gestational age at delivery between the two groups (p < 0.001; Supplementary Fig. S3). The model performed well across all AMSB, with false positive rates ranging between 5.6% (βHCG) and 11.8% (PAPP-A). Internal validation of the model did not significantly alter the performance of the model (Table 3).

**Table 3:** Observer area under the curve (AUC) and optimism adjusted AUC after 1000-fold bootstrapping for adverse outcomes before 34 weeks’ gestation.

Adverse pregnancy outcome < 34 weeks	Original sample			Bootstrapped sample		
	AUC	SE	95% CI	AUC	SE	95% CI
FGR (3rd centile)	0.934	0.033	0.867–1.000	0.950	0.013	0.924–0.976
SGA (< 10th centile)	0.904	0.035	0.835–0.973	0.922	0.018	0.886–0.958
Delivery < 34 weeks	0.816	0.039	0.740–0.892	0.834	0.026	0.784–0.884
Iatrogenic delivery/stillbirth < 34 weeks	0.869	0.040	0.790–0.948	0.841	0.030	0.783–0.899

*AUC* area under curve, *SE* standard error, *CI* confidence interval, *FGR* fetal growth restriction, *SGA* small for gestational age.

There were a small number (17, 1.6%) of screen-negative women who delivered < 34 weeks (Supplementary Table S7). Ten (58.8%) of these were spontaneous preterm births. Two were definite false-negatives with FGR requiring delivery < 34 weeks^10^. These two cases possibly represented EFW measurement error at the placental screen rather than a failure of the model as both had EFW > 15% larger than birthweight, within 3 weeks of delivery. Supplementary Table S8 summarises the causes of the stillbirths, for both positive and negative placental screens.

**Figure 4:**
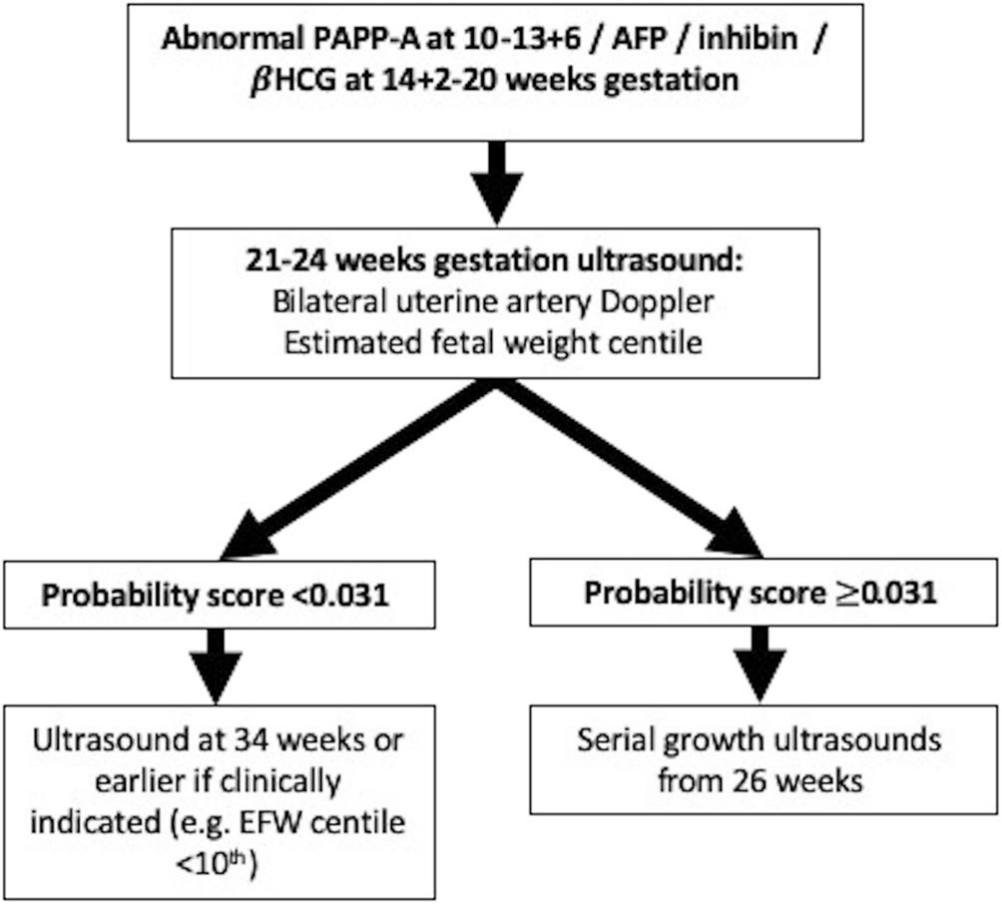
Suggested care algorithm for women with abnormal serum biomarkers (PAPP-A $\le$ 0.415 MoM, βHCG $\ge$ 4.0, MoM, inhibin $\ge$ 2.0 MoM and αFP $\ge$ 2.2 MoM).

Assuming that current common practice would involve three to four weekly scanning from 26 to 28 weeks’ gestation, a minimum of one scan per negative screen could have been avoided by implementing our mid-trimester model and care pathway (Fig. 4). This equates to the avoidance of a minimum of 1044 scans (847 scans per 1000 women with AMSB screened).

The proportion of FGR births < 38 weeks as a proxy for the antenatal detection of FGR has been suggested as a metric within the Saving Babies Lives Care Bundle version 2. In our population cohort 36% of all FGR pregnancies delivered before 38 weeks, 56.9% in those women who had serum screening performed and 65.9% in those who attended for a placental screen.

## Discussion

Our study has confirmed the association between low PAPP-A, and increased βHCG/Inhibin/αFP, with SGA (24.5%) and FGR (10.3%) and demonstrated these markers to be useful incidental pregnancy risk factors when identified through combined aneuploidy screening. This confirms the findings of smaller studies which have reported increased risks of placental disease in women with AMSB^30,31^.

Current Royal College of Obstetricians and Gynaecologists (RCOG) guidance highlights PAPP-A < 0·415 MoM as a risk factor for SGA^28^, but in the current cohort we have confirmed that the risk of FGR was similarly increased for abnormal levels of αFP, inhibin and βHCG. The absence of guidance from current care pathways regarding these additional markers could result in cases of FGR remaining undetected. Given the significantly increased rate of FGR in women with AMSB, third trimester fetal surveillance is justified with the aim of preventing avoidable stillbirths attributable to placental insufficiency through obstetric intervention^11^. We have demonstrated that a combination of two continuous variables (EFW centile and mean uterine artery PI) at 21–24 weeks can effectively rule-out FGR requiring intervention before 34 weeks (NPV 99.8%); a serious, but rare adverse outcome in women with AMSB (2.3% in our cohort) whilst correctly identifying 93% of cases. Uterine artery Doppler PI and EFW centile were the strongest predictors of early-onset FGR in our cohort in agreement with previous findings^32^. Consistent with a recent review by Kingdom et al.^27^, placental biometry was a significant predictor of early-onset FGR, however addition of this to the model did not significantly increase the performance.

Using the combined “placental screen” we suggest that subsequent third trimester ultrasound surveillance can be effectively triaged, such that fetal growth assessment can be safely deferred until after 34 weeks in women with a “negative screen”. In this way, care can be effectively triaged and unnecessary intervention potentially reduced^29^. We have developed an online risk calculator, derived from the internally validated regression model in this study, to simplify decision making at the time of the placental screen: https://drive.google.com/open?id=1v2woSTq7KHNmNDNQ1jHJjv2yUkQ0O7sqfav_NkI_g9Y.

This model, derived from easily attainable 2-dimensional ultrasound measurements, identifies women at risk of FGR requiring intervention before 34 weeks. By adopting the proposed model and care pathway, scan frequency could be reduced for the majority of women (87% had a negative screen in this cohort), with significant cost and time-saving implications for clinicians and patients. Additionally, amongst those with a positive screen, 34 (22%) required delivery < 34 weeks. Without routine surveillance, these pregnancies would have been at very high risk of ending in stillbirth.

This study used previously published thresholds of AMSB to identify a high-risk cohort. The data collated for this study has demonstrated that the cut-offs applied are applicable to our local population in terms of overall screening performance for the detection of FGR. Review of the distribution of PAPP-A measurements, however, would suggest that in our population lowering the cut-off to 0.39 (representing the 5th centile for the SMH population) would increase specificity without compromising sensitivity. Using this threshold requires 51 “placental screens” to be performed per early FGR case detected (see Supplementary Table S2). The thresholds used in our cohort for screening Inhibin and αFP AMSB are more stringent than those applied to PAPP-A and consequently have higher positive predictive values with only 29 and 14 screens being performed per early FGR case detected. Further refining of the population to whom the screen is applied by lowering the threshold at which we offer “placental screens” in this group of women so that equivalent numbers of screens are performed per case detected should be associated with an overall improvement in detection.

Model performance overall will also be influenced by the background prevalence of FGR. In our local population, the prevalence of FGR was 7% and SGA 19%; higher than might be expected and perhaps reflecting the high level of deprivation in our local population. However, FGR and SGA in our hospital population dataset were classified without maternal characteristic customisation due to missing data. As customisation amongst Asian women under classifies SGA, relative to population centiles^33^, it is likely that the prevalence would be lower if customisation were applied.

Study strengths include prospective data collection, exclusion of aneuploid pregnancies, internal validation of the model and a sample size sufficient to assess FGR (< 3rd centile/< 10th centile with absent EDF) rather than SGA (< 10th centile). Despite this being the largest study investigating AMSB in early-onset FGR to date, the most significant limitation of our study was the low primary event rate which reflects the rarity of early-onset FGR. Our model will be inevitably over-fitted to the current cohort, but to minimise the risk of over interpretation we limited the number of included variables to two and performed internal validation, which did not demonstrate a significant shift in model performance. A further limitation is that the clinicians managing the cases were not blinded to the placental screen and local protocol-driven management, based on AMSB, could have altered observed outcomes in this cohort. The severity of AMSB or abnormal ultrasound findings may have impacted on surveillance frequency and therefore timing of delivery. However, we would argue that in practice, knowledge of the placental screen would be unlikely to influence the decision for an indicated preterm delivery, as this was dictated by standard fetal assessments immediately prior to delivery. Furthermore, a placental screen was only performed in pregnancies where AMSB were identified through combined screening and therefore the population studied is limited to those women who chose aneuploidy screening (just under half of the population in this hospital). Whilst there is no indication that the performance of AMSB and a placental screen would be different in a wider obstetric population, it was not possible to confirm this in the current study. The lack of routine placental histology in this cohort limits our ability to correlate the placental screen with distinct placental causes of FGR (i.e. maternal vascular malperfusion (MVM) versus alternative abnormalities (e.g. chronic histiocytic intervillositis) associated with normal uterine artery Dopplers^34^). A positive placental screen and subsequent ultrasound surveillance has the potential to improve perinatal outcomes in early-onset FGR cases through altered obstetric management, highlighted by the fact that 77% (n = 24) of iatrogenic deliveries < 34 weeks indicated for placental disease had a positive screen. In addition, there was a high prevalence of FGR (25%, n = 32) and preterm birth before 37 weeks (28%, n = 36) amongst those with a positive screen, indicating that those with an abnormal assessment at 21–24 weeks are a high-risk group that would benefit from increased surveillance. This study has also highlighted the limitations of second trimester ultrasound in predicting FGR developing near term and emphasised the importance of continued efforts to improve the detection and management of late FGR in high risk women. In our cohort, whilst the detection of FGR (assessed by the number of pregnancies delivered by 38 weeks) was increased in women who had a placental screen in comparison to the SMH population (66% vs 36%), despite ultrasound surveillance, a significant proportion of FGR pregnancies remained undetected.

Placental production of angiogenic markers (including placental growth factor (PlGF) and soluble fms-like tyrosine kinase-1 (sFlt)) is dysregulated in the context of placental dysfunction^35^. For this reason, they are increasingly recognised as diagnostic adjuncts for pre-eclampsia and FGR^36,37^. Additionally, there is evidence to support their predictive role in placental FGR^38,39^, indicating that angiogenic markers could be a useful adjunct to the placental screen. This is beyond the scope of this study, but would be worth investigating in the future, along with newer placental biomarkers^40,41^ with a view to further refining the model.

In conclusion, AMSB are significant risk factors for FGR and monitoring fetal growth in the third trimester is justified with the aim of avoiding preventable stillbirths through earlier obstetric intervention. The majority of FGR in women with AMSB however does not require intervention before 34 weeks; therefore, a “placental screen” at 21–24 weeks can safely reduce scan frequency by ruling out the risk of early-onset FGR in this cohort. A suggested screening model to guide the frequency of fetal surveillance for all AMSB is presented in Fig. 4. By adopting the proposed model and care pathway, scan frequency could be reduced for the majority of women (87% had a negative screen in this cohort). These findings have significant cost and time-saving implications for health services.

## Methods

This retrospective observational cohort study was performed in a single tertiary UK centre between June 2010 and December 2018 using prospectively collected maternal demographic and ultrasound data. Comparison biomarker screening data and birth outcome data for the study period was extracted from the electronic records for pregnancies over the same time period (estimated delivery dates January 2011–December 2018). Only pregnancies with a complete pregnancy outcome, > 22 weeks’ gestation were included in the analysis. Analysis of routinely collected data without the need for individual consent or ethical committee review was nationally approved by the Health Research Authority (HRA; 19/HRA/2047) and locally by Manchester University NHS Foundation Trust (MFT) Research and Innovation. The study has been reported in line with the STROBE guidance for reporting in observational studies^42^. Biomarker measurements were performed as part of routine fetal chromosomal abnormality screening between 11 and 13 + 6 weeks’ gestation (PAPP-A), and 14 and 17 + 6 weeks’ gestation (beta human chorionic gonadotropin (βHCG), inhibin, and alpha fetoprotein (αFP)). Biomarker concentrations were reported by the laboratory as standard multiples-of-median (MoM) corrected for gestational age^43^.

**Figure 5:**
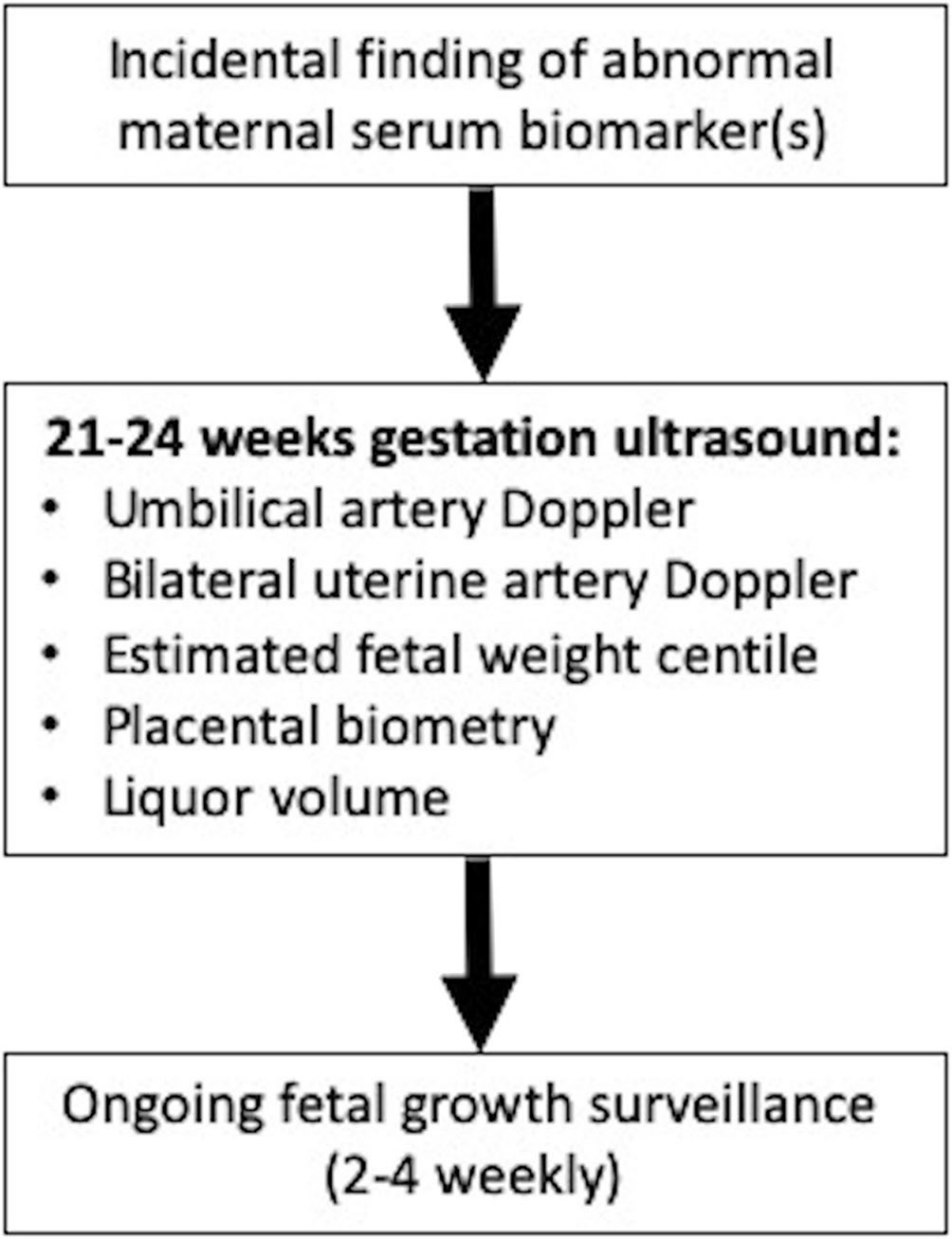
Manchester University NHS Foundation Trust (MFT) Placenta Clinic referral pathway.

As per local guidance (Fig. 5), women at increased risk of FGR were referred to the Placenta Clinic and Manchester Antenatal Vascular Service (MAViS Clinic), specialist translational research clinics (LREC No. 08/H1010/55+5; 15/NW/0929; 11/NW/0426). Referral criteria include an incidental finding of AMSB (PAPP-A $\le$ 0.415 MoM (5th centile)^10,12,44^, βHCG $\ge$ 4.0 MoM^12,44,45^, inhibin $\ge$ 2.0 MoM^4,12,44^ and αFP $\ge$ 2.2 MoM^4,12,44^). In this clinic, women undergo a 21–24 week placental screen, in which liquor volume (amniotic fluid index and maximum pool depth), placental and fetal biometry, and umbilical and uterine artery Dopplers are measured. During the study period, the scan at 21–24 weeks did not trigger intervention or alter the frequency of surveillance although the findings were reported to the clinicians.

Placental biometry was measured using the following method^26^: the longest plane of the placenta was identified using 2-dimensional ultrasound. The placental diameter was then measured (end-to-end) using one or two adjoining straight lines. Placental depth was measured at the deepest point, perpendicular to its diameter. Following 90° rotation of the ultrasound probe, the second diameter was measured (end-to-end, using one or two adjoining straight lines).

As per our routine clinical practice, customised birthweight centiles^46^ were used to calculate both the EFW centile and final birthweight centile in the cohort study. A sensitivity analysis included performance of the model for early-onset FGR defined using non-customised centile. SGA was defined as < 10th centile birthweight and FGR was defined as < 3rd centile birthweight/< 10th with absent end-diastolic flow (EDF). Early-onset FGR was defined as an fetus requiring delivery before 34 weeks’ gestation with birthweight < 3rd centile or < 10th centile with absent EDF. Due to missing data for maternal ethnicity, parity and body mass index in the hospital electronic records, birthweight centiles in the population dataset were calculated without customisation (using Hadlock).

### Statistical methods

The distribution of continuous variables was assessed for normality using the Jarque–Bera skewness-kurtosis test and data appropriately transformed. Chi-squared test was used to compare categorical variables between the two groups. The association between each of the ultrasound variables and FGR was assessed using univariate comparisons. STATA version 14.2 was used to derive a logistic regression model restricted to three variables (to avoid overfitting) to determine the accuracy of prediction for early-onset (< 34 weeks’) FGR. Different combinations of variables were included in the model; the performance of each model was then determined using receiver operator characteristics (ROC) curve analyses. These areas were compared using DeLong method to determine the best model. Due to non-normality of uterine artery PI and EFW, these variables were log transformed. Continuous variables were compared between test-positive and test-negative women using t test/Mann–Whitney as appropriate. Varying probability cut-offs were tested to determine the optimum positive and negative likelihood ratios for the regression model. The models were subjected to a bootstrapping sample, with replacement from the same dataset with 1000 replications. Model performance (AUC, 95% CI) was compared between the original and bootstrap samples. The coefficients for each variable in the final regression model were used to create a web-based risk prediction calculator.

## Supplementary Information

Supplementary Information.
