# Supplementary material for: Risk stratification for early-onset fetal growth restriction in women with abnormal serum biomarkers: a retrospective cohort study
Source: Sci Rep. 2020 Dec 17;10:22259. doi: 10.1038/s41598-020-78631-5 (PMC7746767; doi:10.1038/s41598-020-78631-5)
Supplement: Supplementary file 1 — Supplementary Information. [file 41598_2020_78631_MOESM1_ESM.docx]

**Risk stratification for early-onset fetal growth restriction in women with abnormal serum biomarkers: a retrospective cohort study.**

Ormesher L^1,2^, Warrander L.^1,2^, Liu, Y.^3^, Thomas S.^2^, Simcox L.^2^, Smith GCS^4^, Myers JE.^1,2^, Johnstone ED. ^1,2^.

1. Division of Developmental Biology and Medicine, School of Medical Sciences, Faculty of Biology, Medicine and Health, University of Manchester, Manchester Academic Health Science Centre, Manchester, UK

2. St Mary’s Hospital, Central Manchester University Hospitals NHS Foundation Trust, Oxford Road, Manchester, UK

3. Monash University, Scenic Boulevard & Wellington Road, Clayton 3800, Australia

4. Department of Obstetrics and Gynaecology, University of Cambridge, Cambridge, UK

5. NIHR Cambridge Biomedical Research Centre, Cambridge, UK

Corresponding author: Professor Edward Johnstone

MFHRC, 5^th^ Floor St Mary’s Hospital, Central Manchester University Hospitals NHS Foundation Trust, Oxford Road, Manchester M13 9WL.

0161 701 6960 / Edward.johnstone@manchester.ac.uk

**Supplementary table 1: Population prevalence of adverse pregnancy outcomes**

|  | **N** | **%** | **95% C.I.** |
| --- | --- | --- | --- |
| SMH births (2011-2018) | 67065 |  |  |
| Total serum screening results | 29796 | 44.4% |  |
| Combined | 20846 | 67.0% |  |
| Second Trimester | 8950 | 30.0% |  |
| SMH Births with outcome >22 weeks | 65192 |  |  |
| Live Birth | 64729 | 99.3% |  |
| Perinatal Death | 436 | 0.7% | 0.6-0.7 |
| FGR (<3rd Centile) | 4491 | 6.9% | 6.7-7.1 |
| SGA (<10th Centile) | 12355 | 19.0% | 18.6-19.2 |
| Birth <34 weeks | 1582 | 2.4% | 2.4-2.6 |
| eFGR | 427 | 0.67% | 0.6-0.7 |

C.I., confidence interval; SMH, St Mary’s Hospital; EDD, estimated delivery date; FGR, fetal growth restriction; SGA, small for gestational age; eFGR, early-onset fetal growth restriction

**Supplementary Table 2: Performance of maternal serum biomarker MoM thresholds for the prediction of FGR and early-onset FGR in SMH hospital population 2011-2018.**

|  |  | | **All FGR** | | | | | **eFGR** | | | | | | | |
| --- | --- | --- | --- | --- | --- | --- | --- | --- | --- | --- | --- | --- | --- | --- | --- |
| **Threshold** | **Cut-off** | | **AUC/prevalence** | **Sens** | **Spec** | **PPV** | **NPV** | **AUC/prevalence** | **Sens** | **Spec** | **PPV** | **NPV** | **Screen +ve 10,000^*^** | **Number of eFGR detected**† | **Number needed to screen to detect 1 case**‡ |
|  | | **PAPP-A** | | | | | | | | | | | | | |
| **<5th**(1–3) | **0.415** | | **0.602**  **1007/17882 (5.6%)** | 13.3 | 94.4 | 12.4 | 94.8 | **0.657**  **79/17882 (0.4%)** | 25.3 | 94 | 1.84 | 99.65 | 609 | 10/44 | 59 |
| **SMH 5th** | **0.392** | |  | 11.9 | 95.5 | 13.6 | 94.8 |  | 24.0 | 95.1 | 2.13 | 99.65 | 500 | 10/44 | 51 |
| **SMH 3rd** | **0.341** | |  | 8.8 | 97.3 | 16.5 | 94.7 |  | 17.7 | 97.1 | 2.65 | 99.63 | 300 | 7/44 | 41 |
|  | | **Inhibin** | | | | | | | | | | | | | |
| **SMH 95th (current)**(1,3,4) | **2.03** | | **0.547**  **559/7628 (7.3%)** | 10.9 | 95.4 | 15.4 | 93.3 | **0.737**  **52/7628 (0.7%)** | 27.6 | 95.1 | 3.68 | 99.49 | 500 | 17/67 | 29 |
| **SMH 97th** | **2.31** | |  | 7.8 | 97.3 | 17.8 | 93.2 |  | 20.7 | 97.1 | 4.6 | 99.45 | 300 | 13/67 | 23 |
| **SMH 99th** | **3.03** | |  | 4.2 | 99.3 | 30.2 | 93.1 |  | 13.8 | 99.1 | 9.3 | 99.42 | 87 | 9/67 | 10 |
|  | | **αFP** | | | | | | | | | | | | | |
| **SMH 95th** | **1.74** | | **0.575**  **561/7629 (7.4%)** | 11.8 | 95.5 | 16.9 | 93.4 | **0.705**  **52/7629 (0.7%)** | 22.4 | 95.1 | 3 | 99.45 | 500 | 14/67 | 36 |
| **SMH 97th** | **1.90** | |  | 8.8 | 97.4 | 20.8 | 93.3 |  | 19.0 | 97.1 | 4.23 | 99.44 | 300 | 12/67 | 25 |
| **Current**(1,3,4) | **2.2** | |  | 4.4 | 98.7 | 20.5 | 93.1 |  | 15.5 | 98.57 | 6.82 | 99.43 | 133 | 10/67 | 14 |

^*^Theoretical population of 10,000 screened women.

†Based on SMH prevalence of eFGR of 44/10000 in women with a 1^st^ Trimester screen & 67/10000 in women having a 2^nd^ Trimester screen, and assuming all women attend a placental screen with a detection rate of 93% for eFGR (estimated fetal weight and uterine artery Doppler at 21-24 weeks).

‡Number of placental screen scans (21-24) weeks which would need to be performed to detect 1 case of eFGR.

FGR, fetal growth restriction; eFGR, early-onset fetal growth restriction; AUC, area under the curve; Sens, sensitivity; Spec, specificity; PPV, positive predictive value; NPV, negative predictive value; SMH, St Mary’s Hospital; PAPP-A, pregnancy associated plasma protein-A, αFP, alpha fetoprotein.

**Supplementary Table 3: Univariate associations between baseline characteristics and 21-24 week ultrasound findings for early-onset FGR.**

| **Variable** | **N** | **P** | **OR** | **95% C.I.** |
| --- | --- | --- | --- | --- |
| Customised EFW centile†  (increment 5 centiles) | 1196 | <0.001* | 0.66 | 0.58-0.75 |
| Non-customised EFW centile  (increment 5 centiles) | 1196 | <0.001* | 0.63 | 0.54-0.73 |
| Umbilical artery PI (increment 0·1) | 1186 | <0.001* | 1.98 | 1.55-2.51 |
| Umbilical artery RI  (increment 0·1) | 1182 | <0.001* | 7.08 | 3.06-16.26 |
| Uterine artery PI†  (increment 0·1) | 1196 | <0.001* | 1.47 | 1.34-1.60 |
| Uterine artery RI  (increment 0·1) | 1196 | <0.001* | 6.48 | 3.93-10.70 |
| Placental depth | 1196 | 0.001* | 1.76 | 1.27-2.44 |
| Placental surface area | 1196 | <0·001* | 0.97 | 0.96-0.98 |
| PEC (width x width / depth) | 1196 | <0·001* | 0.93 | 0.91-0.96 |
| Maternal BMI | 914 | 0.848 | 1·01 | 0.92-1.10 |
| Maternal sBP at first prenatal visit | 1135 | 0.031* | 1·03 | 1.00-1.06 |
| Maternal dBP at first prenatal visit | 1132 | 0.045* | 1.04 | 1.00-1.09 |
| Maternal ethnicity  (compared to white)  Black  Asian  Other | 1196  692  159  211  134 | 0.606  0.220  0.782 | 1.35  1.79  1.20 | 0.43-4.19  0.71-4.55  0.34-4.26 |
| Maternal parity (nullip vs multip) | 1196 | 0.335 | 0.68 | 0.31 – 1.49 |

OR, odds ratio; C.I., confidence interval; EFW, estimated fetal weight; PI, pulsatility index; RI, resistance index; PEC, placental efficiency coefficient; BMI, body mass index; sBP, systolic blood pressure; dBP, diastolic blood pressure.

*Statistically significant

†Retained for use in the multivariable model

**Supplementary Table 4: 21-24 week placental screen test performance for early-onset FGR, with and without PSA**.

|  | **Without placental surface area** | | |
| --- | --- | --- | --- |
|  | **True +ve** | **True -ve** |  |
| **Test +ve** | 25 | 127 | **PPV=16.5%** |
| **Test -ve** | 2 | 1042 | **NPV=99.8%** |
|  | **Sensitivity=92.6%** | **Specificity=89.1%** |  |
|  | **+LR = 8.53** | **-LR = 0·08** |  |
|  | **With placental surface area** | | |
|  | **+ve** | **True -ve** |  |
| **Test +ve** | 26 | 120 | **PPV=17.8%** |
| **Test -ve** | 1 | 1049 | **NPV=99.9%** |
|  | **Sensitivity=96.3%** | **Specificity=89.7%** |  |
|  | **+LR = 9.38** | **-LR = 0.04** |  |

PPV, positive predictive value; NPV, negative predictive value; +LR, positive likelihood ratio; -LR, negative likelihood ratio.

**Supplementary Table 5: Multivariable logistic regression coefficients for 1196 women who underwent a 21-24 week placental screen.**

|  | **Coefficient** | **Standard Error** | **z** | **P** | **95% C.I.** |
| --- | --- | --- | --- | --- | --- |
| **Log mean uterine artery PI** | 4.39 | 0.77 | 5.66 | <0.001 | 2.87 – 5.91 |
| **Log EFW centile** | -0.71 | 0.17 | -4.24 | <0.001 | -1.04 – -0.38 |
| **Constant** | -2.08 | 0.66 | -3.17 | 0.002 | -3.37 – -0.79 |

C.I., confidence interval; PI, pulsatility index; EFW, estimated fetal weight; PSA, placental surface area.

**Supplementary Table 6: Prevalence of adverse pregnancy outcomes in the test-negative and test-positive groups**

| **Outcome** | **Negative placental screen (n=1044)** | **Positive placental screen (n=152)** |  |
| --- | --- | --- | --- |
|  | **N (%)** | **N (%)** | **OR [95% C.I.]** |
| **SGA <10^th^ centile** | 203 (19.4%) | 90 (59.2%) | 6.01 [4.20-8.60] |
| **FGR <3^rd^ centile** | 66 (6.3%) | 57 (37.5%) | 8.89 [5.89-13.43] |
| **Preterm <36 weeks** | 57 (5.5%) | 50 (32.9%) | 8.49 [5.52-13.06] |
| **Preterm <34 weeks** | 17 (1.6%) | 34 (22.4%) | 17.41 [9.43-32.12] |
| **Iatrogenic preterm <34 weeks** | 7 (0.7%) | 24 (15.8%) | 27.78 [11.73-65.75] |
| **Preterm FGR (3^rd^ centile) <34 weeks** | 2 (0.2%) | 25 (16.5%) | 102.56 [24.01-438.10] |
| **Stillbirth** | 5 (0.5%) | 7 (4.6%) | 10.03 [3.14-32.02] |
| **Stillbirth < 34 weeks** | 2 (0.2%) | 7 (4.6%) | 25.15 [5.18-122.24] |

OR, odds ratio; C.I., confidence interval; SGA, small for gestational age; FGR, fetal growth restriction.

**Supplementary Table 7: Details of the pregnancies with false negative placental screens (n=2) and those who had negative screens who delivered <34 weeks >3^rd^ centile**

| **Mean uterine artery PI** | **EFW centile** | **Abnormal maternal serum biomarker** | **Birth gestation (weeks + days)** | **BW centile** | **Indication for delivery** | **Placental histology** |
| --- | --- | --- | --- | --- | --- | --- |
| 1.06 | 22.2 | PAPP-A | 26+3 | 0.0 | Elective Caesarean Section for absent umbilical artery EDF, static growth and abnormal NST | No histology available |
| 0.65 | 88.6 | βHCG | 25+3 | 0.7 | Emergency Caesarean Section for placental abruption; EFW at placental screen was incorrect | No histology available |
| 0.69 | 0.7 | PAPP-A | 32+0 | 4.6 | Elective Caesarean Section for abnormal umbilical artery Dopplers and reduced fetal movements | MVM |
| 0.68 | 29.0 | βHCG | 30+2 | 4.6 | Premature rupture of membranes and abnormal NST | No histology available |
| 1.16 | 24.9 | PAPP-A | 32+4 | 5.8 | Stillbirth following placental abruption | Focal area of CHIV; evidence of abruption; no MVM. |
| 0.99 | 49.3 | PAPP-A | 32+2 | 6.1 | Spontaneous preterm labour | No histology available |
| 0.85 | 32.4 | PAPP-A | 33+2 | 9.1 | Spontaneous preterm labour; large fibroid | No histology available |
| 0.56 | 64.5 | PAPP-A | 33+2 | 14.3 | Spontaneous preterm labour | No histology available |
| 1.09 | 86.9 | PAPP-A | 23+5 | 16.5 | Spontaneous preterm labour | No histology available |
| 0.91 | 51.0 | PAPP-A | 33+2 | 17.4 | Planned Caesarean Section due to absent umbilical end diastolic flow | Inconclusive - possible CHIV or possible MVM |
| 1.07 | 59.0 | Inhibin | 32+5 | 19.2 | Planned Caesarean section for pre-eclampsia; normal fetal growth trajectory | MVM |
| 0.65 | 61.4 | PAPP-A | 33+2 | 20.6 | Normal fetal growth trajectory; stillbirth 1 week after a follow-up scan | CHIV |
| 0.77 | 13.4 | Inhibin | 25+0 | 27.2 | Spontaneous preterm labour | CHIV |
| 1.11 | 80.8 | PAPP-A | 31+6 | 46.9 | Spontaneous preterm labour |  |
| 1.05 | 88.5 | Inhibin | 32+4 | 50.1 | Spontaneous preterm labour | No histology available |
| 0.79 | 50.7 | αFP & inhibin | 26+2 | 58.1 | Spontaneous preterm labour | Chorioamnionitis |
| 0.65 | 64.8 | PAPP-A | 32+0 | 100.0 | Spontaneous preterm labour | No histology available |

PI, pulsatility index; EFW, estimated fetal weight; BW, birthweight; βHCG, beta human chorionic gonadotropin; PAPP-A, pregnancy-associated plasma protein-A; αFP, alpha-fetoprotein; EDF, end-diastolic flow; NST, non stress test; MVM, maternal vascular malperfusion; CHIV, chronic histiocytic intervillositis.

**Supplementary table 8: Details of the pregnancies complicated by stillbirth**

| **Placental screen** | **Gestation at birth (weeks + days)** | **Birthweight centile** | **Placental histology** | **Preventable?** | **Other details** |
| --- | --- | --- | --- | --- | --- |
| Positive | 22+6 | 0.0 | Nil available | Not preventable | Mother had CREST syndrome |
| Positive | 27+2 | 0.9 | Nil available | Not preventable | Mother was homozygous for Factor V Leiden |
| Positive | 26+0 | 0.0 | MVM | Not preventable | Mother had antiphospholipid syndrome |
| Negative | 40+3 | 3.9 | Nil available | Possibly preventable | Normal scan at 35+6 weeks; transferred care; no growth after last scan |
| Negative | 32+4 | 5.8 | Focal area of CHIV; no MVM; abruption | Not preventable | Placental abruption |
| Positive | 25+2 | 0.0 | Nil available | Not preventable | - |
| Negative | 37+3 | 54.0 | Nil available | Possibly preventable | Delivered elsewhere |
| Positive | 25+2 | 0.0 | Possible MVM (inconclusive) | Not preventable | - |
| Negative | 42+1 | 8.7 | MVM | Preventable | - |
| Positive | 33+6 | 35.7 | No focal pathology seen | Possibly preventable | - |
| Negative | 33+2 | 20.6 | Fresh haemorrhage; no MVM | Not preventable | Placental abruption |
| Positive | 33+2 | 0.1 | MVM | Possibly preventable | - |

CREST, limited scleroderma; MVM, maternal vascular malperfusion; CHIV, chronic histiocytic intervillositis.

**Supplementary Figure 1: Probability of fetal growth restriction (<3rd centile) generated from univariate analysis of each biomarker.**


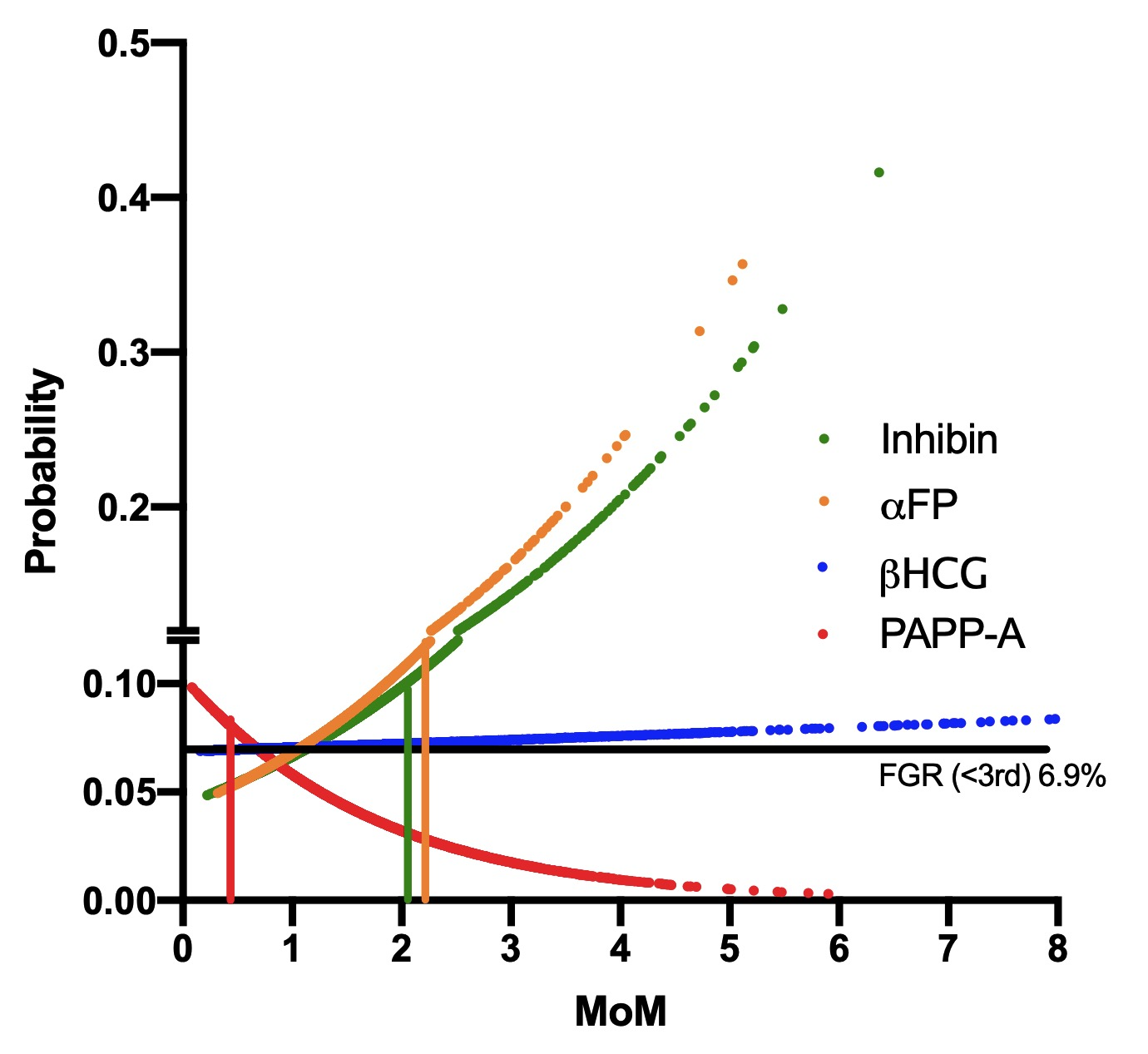


The vertical markers indicate the threshold used to trigger referral for a 21-24 placental screen scan.

αFP, alpha fetoprotein, βHCG, beta-human chorionic gonadotrophin; PAPP-A, pregnancy-associated plasma protein-A; ; FGR, fetal growth restriction; MoM, multiple of the median.

**Supplementary Figure 2: Difference in customised birthweight centile between those who test negative and those who test positive (p<0.001)**

**Supplementary Figure 3: Difference in gestational age at delivery between those who test negative and those who test positive (p<0.001)**

**References**

1. Lakhi N, Govind A, Moretti M, Jones J. Maternal serum analytes as markers of adverse obstetric outcome. Obstet Gynaecol. 2012;14(267–273).

2. Royal College of Obstetricians & Gynaecologists. The Investigation and Management of the Small–for–Gestational–Age Fetus. Green-top Guideline No.31. 2013.

3. Gagnon A, Wilson RD, Audibert F, Allen VM, Blight C, Brock JA, et al. Obstetrical Complications Associated With Abnormal Maternal Serum Markers Analytes. J Obstet Gynaecol Canada. 2008;30(10):918–32.

4. Morris RK, Cnossen JS, Langejans M, Robson SC, Kleijnen J, ter Riet G, et al. Serum screening with Down’s syndrome markers to predict pre-eclampsia and small for gestational age: Systematic review and meta-analysis. BMC Pregnancy Childbirth. 2008;8:33.
